# Genomic Characterization and High Prevalence of Bocaviruses in Swine

**DOI:** 10.1371/journal.pone.0017292

**Published:** 2011-04-15

**Authors:** Tongling Shan, Daoliang Lan, Linlin Li, Chunmei Wang, Li Cui, Wen Zhang, Xiuguo Hua, Caixia Zhu, Wei Zhao, Eric Delwart

**Affiliations:** 1 Zoonosis and Comparative Medicine Group, Shanghai Jiao Tong University, Shanghai, People's Republic of China; 2 Blood Systems Research Institute, San Francisco, California, United States of America; 3 Department of Laboratory Medicine, University of California San Francisco, San Francisco, California, United States of America; 4 College of Veterinary Medicine, Huazhong Agricultural University, Wuhan, Huibei, People's Republic of China; 5 School of Medical Science and Laboratory Medicine, Jiangsu University, Zhenjiang, Jiangsu, People's Republic of China; Tulane School of Public Health and Tropical Medicine, United States of America

## Abstract

Using random PCR amplification followed by plasmid subcloning and DNA sequencing, we detected bocavirus related sequences in 9 out of 17 porcine stool samples. Using primer walking, we sequenced the nearly complete genomes of two highly divergent bocaviruses we provisionally named porcine bocavirus 1 isolate H18 (PBoV1-H18) and porcine bocavirus 2 isolate A6 (PBoV2-A6) which differed by 51.8% in their NS1 protein. Phylogenetic analysis indicated that PBoV1-H18 was very closely related to a ∼2 Kb central region of a porcine bocavirus-like virus (PBo-LikeV) from Sweden described in 2009. PBoV2-A6 was very closely related to the porcine bocavirus genomes PBoV-1 and PBoV2 from China described in 2010. Among 340 fecal samples collected from different age, asymptomatic swine in five Chinese provinces, the prevalence of PBoV1-H18 and PBoV2-A6 related viruses were 45–75% and 55–70% respectively, with 30–47% of pigs co-infected. PBoV1-A6 related strains were highly conserved, while PBoV2-H18 related strains were more diverse, grouping into two genotypes corresponding to the previously described PBoV1 and PBoV2. Together with the recently described partial bocavirus genomes labeled V6 and V7, a total of three major porcine bocavirus clades have therefore been described to date. Further studies will be required to elucidate the possible pathogenic impact of these diverse bocaviruses either alone or in combination with other porcine viruses.

## Introduction

Members of the *Parvoviridae* family are small non-lipid enveloped viruses with a diameter of 18–26 nm, icosahedral symmetry (T = 1), encoded by a single-stranded linear DNA genome of approximately 4,000 to 6,000 nucleotides (nt) [1]. The family includes two subfamilies: *Densovirinae*, *Parvovirinae*. The subfamily of *Densovirinae* contains four genera: *Densovirus*, *Iteravirus*, *Brevidensovirus* and *Pefudensovirus*, which infect only invertebrates [1]. The *Parvovirinae* subfamily is currently subdivided into five genera: *Parvovirus*, *Erythrovirus*, *Dependovirus (adeno-associated virus)*, *Amdovirus*, and *Bocavirus*, infecting vertebrates [1].

The bocavirus genus was recently assigned by the International Committee on Taxonomy of Viruses (ICTV) [1] to parvovirus genomes containing a third ORF (labeled NP1) between the NS1 and VP1/VP2 genes [2]. Bocaviruses were first identified in bovine and canine [3], [4], samples from which it derives its genus name [1], [5]. Presently, the bocavirus genus contains eight members: bovine parvovirus, canine minute virus (CnMV), human bocavirus 1–4 (HBoV1-4), a gorilla bocavirus and a partially sequenced chimpanzee bocavirus [1], [6], [7].

The first human bocavirus (HBoV) was found in the nasopharyngeal secretion of a child with respiratory problems using a methodology closely related to that used here [8]. HBoV has been associated with lower respiratory tract symptoms and possibly diarrhea [5], [9]–[22], and shows a very low degree of genetic variability worldwide [6], [23], [24]. HBoV2 was first reported in the stool of Pakistani children with non-polio acute flaccid paralysis (AFP) [10], and then in Australian children and Chinese children with diarrhea [9], [25]. HBoV3 was first reported in the stool of Australian children with diarrhea [9] and then in stool from Nigerian, Tunisian, Nepalese and US children [5]. HBoV4 was reported in the stool of children with AFP from Nigeria and Tunisia [5]. HBoV 1/2/3 were also detected in untreated sewage water from throughout the US [26]. Recently, a novel bocavirus was identified in the feces of captive gorillas with diarrhea [6] and from wild gorillas and chimpanzees [7]. In 2009, a porcine boca-like virus (PBo-likeV) was reported in swine feces with postweaning multisystemic wasting syndrome in Sweden and 1854 bp of its partial genome sequenced [27]. In 2010, the nearly complete genomes of distinct porcine bocaviruses provisionally named PBoV1 and PBoV2 were characterized from feces of swine in China [28]. Finally, partial genome sequences of 2.4 Kb from another clade of porcine bocaviruses labeled 6V and 7V where also identified yielding three major bocavirus groups in swine (PBo-likeV, PBoV1/PBoV2, and 6V/7V).

Random amplification and sequencing has been used to discover novel virus in human and animal [8], [10], [29]–[53]. In this study, we found in swine feces highly distinct bocavirus whose genome we tentatively named porcine bocavirus 1 (PBoV1-H18), and porcine bocavirus 2 (PBoV 2-A6). The nearly complete genomes of both viruses were acquired and are described here. PBoV1-H18 and PBoV2-A6 were also screened for in 340 stool samples of asymptomatic swine from five provinces of China.

## Materials and Methods

### Sample collection

A total of 340 porcine stool samples from different aged swine were collected from 17 middle or large-scale porcine farms (200–2,000 sows each) in five provinces of China from April 2008 to October 2009, of which 80 were collected from four farms located in Shanghai, 60 from three farms located in Jiangsu province, 120 from six farms located in Anhui province, 20 from a farm located in Shandong province, and 60 from three farms located in Guizhou province and stored at −80°C (Table 1).

**Table 1:** High prevalence of PBoV1-H18 and PBoV2-A6 detected by nested–PCR assay in fecal samples from different aged pigs in five provinces of China.

Location(province)	Number of farms	Age(day)	Positive/tested samples for PBoV1-H18	Positive/tested samples for HBoV2-A6	Co-infection/tested samples
Shanghai	**4**	<45	22/40	24/40	
		45–90	27/40	23/40	
			49/80 (61.3%)	47/80 (58.8%)	31/80 (38.8%)
Jiangsu	**3**	<45	10/30	24/30	
		45–90	18/30	21/30	
			35/60 (58.3%)	41/60 (68.3%)	22/60 (36.7%)
Anhui	**6**	<45	41/60	42/60	
		45–90	36/60	39/60	
			77/120 (64.2%)	81/120 (70.1%)	46/120 (38.3%)
Shandong	**1**	<45	5/10	5/10	
			4/10	6/10	
			9/20 (45%)	11/20 (55%)	6/20 (30%)
Guizhou	**3**	<45	26/30	21/30	
		45–90	19/30	18/30	
			45/60 (75%)	39/60 (65%)	28/60 (46.7%)
Total	**17**		215/340 (63.2%)	219/340 (64.4%)	133/340 (39.1%)

### Viral particle purification and RT-PCR

One stool sample was randomly selected from each of the 17 farms. The samples were suspended in PBS (0.01 M phosphate, pH7.2–7.4, 0.15NaCl), vortexed, centrifuged at 15000 g, and filtered through a 0.22-µm filter to remove eukaryotic- and bacterial-cell-sized particles [45], [46], [54], [55]. The filtrates were then treated with benzonase, DNase and RNase to digest non-particle-protected nucleic acid as reported [54], [55]. Viral nucleic acids were then extracted using the TIANamp virus DNA/RNA Kit (TIANGEN BIOTECH, BEIJING, CO., Ltd.). Viral cDNA synthesis was performed by incubation of the extracted viral RNA/DNA with 100 pmol of primer K-8N [56] with a degenerate 3′ end and the use of Superscript reverse transcriptase, and the opposite strand of the cDNA was generated after melting and reannealing and primer extension using Klenow DNA polymerase [45], [46], [54]–[56]. PCR of extension products was performed as reported previously using the K-8 primer (K-8N without the degenerate 3′ end) [54]. This protocol amplifies both viral RNA and DNA genomes [54], [55].

### Novel virus identification and complete genome sequencing

Random RT-PCR DNA products ran as smears on agarose gel and were gel purified (Axygen, CA, USA), then subcloned into pMD-18T plasmid vector (TaKaRa, Japan) for sequencing. The sequences were then screened for sequence similarities using tBLASTx and BLASTn against the nr database in GenBank.

### Molecular epidemiology

DNA and RNA were also directly extracted from centrifuged stool supernatant using the TIANamp virus DNA/RNA Kit (TIANGEN BIOTECH, BEIJING, CO., Ltd.). For PBoV1-H18 related sequences screening, primers PBoV1-L1 (5′-CTGTGGCACTTCAGATTTAC-3′) and PBoV1-R1 (5′-TCTGTTTTGTGTATTTGTGG-3′) were used for the first round of nested PCR, and the primers PBoV1-L2 (5′-ACAGGAATTAACAGACGAAG-3′) and PBoV1-R2 (5′-TATCGGCACGTACCATTGAC-3′) were used for the second round of nested PCR, resulting in the amplification of a 530-bp fragment over the NP1 and VP1 genes. PBoV2-A6 related sequences were detected using primers PBoV2-L1 (5′-CAAGGGCGCTACACACACAA-3′) and PBoV2-R1 (5′-TTAATTCCGCACTTAGTTGG-3′) for the first round of nested PCR; primers PBoV2-L2 (5′-TCCAGTAACCAAAACATACC-3′) and PBoV2-R2 (5′- TCTCGTGTTGATTGTAGCTC-3′) for the second round of nested PCR, amplifying a 439-bp fragment of the VP1 gene.

### Sequence and phylogenetic analysis

Sequences of each PCR product were assembled using SeqMan II program (DNASTAR, Inc). The identification of open reading frames (ORFs) was performed by a translated BLAST search (BLASTx at http://www.ncbi.nlm.nih.gov/blast/Blast.cgi) and an ORF finder at the website (http://www.ncbi.nlm.nih.gov/gorf/gorf.html). Sequences used for the comparison were comprised the following: HBoV1-4 (DQ000495, NC_007455, EU918736, GQ867667, FJ973561, FJ375129, FJ170278 and NC_012042), GBoV1 (HM145750), CnMV (AB158475, FJ899734, AF495467 and FJ214110), BPV1 (DQ335247 and NC_001540), canine parvovirus (D26079, NC_001539 and EU310373), porcine parvovirus (EU790642), mice minute virus (J02275), mouse parvovirus 1(NC_001630), bovine parvovirus (NC_006259), porcine parvovirus 4 (HM031135, GQ387500, GQ387499), human parvovirus B19 (FJ591158), simian parvovirus (U26342), human parvovirus 4 (AY622943), goose parvovirus (NC_001701), Muscovy duck parvovirus (NC_006147), PBo-likeV (FJ872544), PBoV1 and PBoV2 (HM053693 and HM053694), and 6V and 7V (HM053672 and HM053673). Multiple sequence alignment was performed using CLUSTAL W. Protein amino acid distances were calculated using the MegAlign program (DNASTAR, Inc). Phylogenetic trees were generated using the neighbor joining (NJ) method with bootstrap of 1,000 replicates with MEGA 4.1 (http://www.megasoftware.net/mega41.html).

### Nucleotide sequence accession numbers

The near-full genomes of PBoV1-H18, PBoV2-A6 and the partial NS1 and VP1 sequences from the diagnostic nPCR have been deposited in GenBank under accession numbers HQ291308-HQ291309 and HQ291310-HQ291343.

## Results

### Novel porcine bocavirus sequence

Seventeen porcine samples stool supernatants from 17 farms were analyzed using a generic viral particle-protected nucleic acid enrichment procedure followed by random amplification of extracted RNA and DNA (see materials and methods) [46], [54]–[56]. Amplified DNA was then subcloned and 1190 plasmid inserts were sequenced (70 for each of 17 samples). Nine samples (totaling 82 subclones) showed the presence of fragments whose virtual translation products were related to canine and human bocaviruses using BLASTx. Twenty-four clones from pig sample H18 and 26 clones from pig sample A6 showed significant similarity with bocaviruses. H18 derived sequences showed high (>99%) identity with the recently described porcine bocavirus-like virus (PBo-LikeV), the only porcine bocavirus reported at the time of these experiments (GenBank GU902971) [27]. Sequences from porcine sample A6 showed low identity with those of H18 and PBo-likeV. The H18 and A6 samples were selected for targeted viral genome amplification and sequencing.

### Nearly complete genomes of PBoV1 and PBoV2

The 24 sequences from H18 were assembled to form a continuous sequence of approximately 2700 nucleotides that appeared to lack >1300 and 1000 nucleotides from the 5′and 3′ ends of its genome. PCR primers based on the available H18 sequences and regions highly conserved between HBoV2 (FJ170278) and canine bocavirus (FJ214110) were used to amplify the nearly complete bocavirus genome we provisionally called PBoV1-H18 (5267 nt). To confirm this genome sequence, this sequence was re-amplified using 6 sets of PCR primers generating overlapping fragments of the genome which were directly sequenced. Using the same method, the nearly complete bocavirus genome (5117 nt) from sample A6 was also sequenced and was provisionally labeled PBoV2-A6.

**Figure 1:**
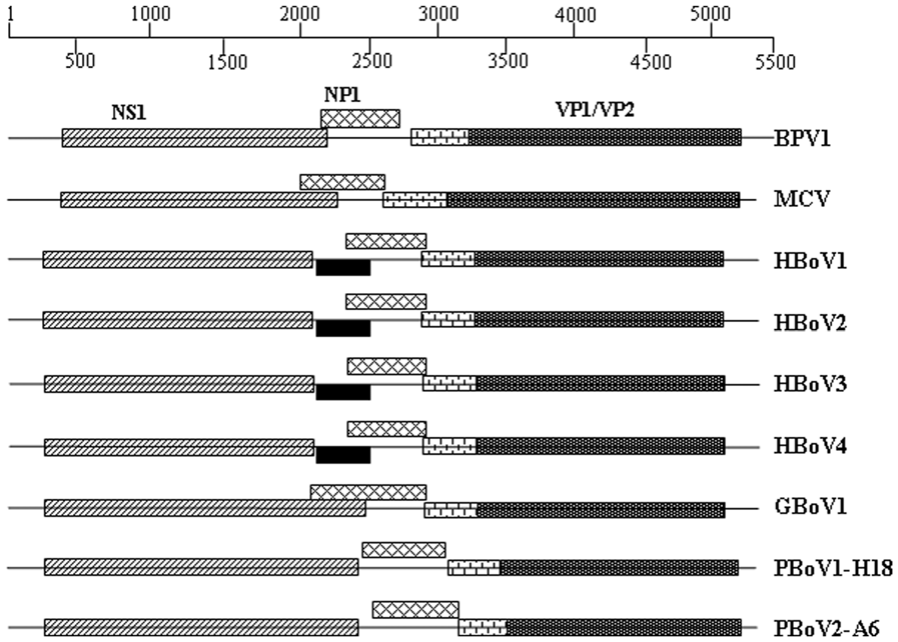
The PBoV1 and PBoV2 genomes. Diagrammatic representation of PBoV1-H18 and PBoV2-A6 sequences showing the position of ORFs for NS1, NP1, VP1 and VP2, compared with other bocaviruses. The black boxes represent the known spliced exon of NS1 transcripts.

Using an open reading frame (ORF) finder (http://www.ncbi.nlm.nih.gov/gorf/gorf.html), three ORF were found in both genomes (Figure 1). The ORFs of PBoV1-H18 were 636 aa for NS1, 219 aa for NP1 and 621 aa for VP1/VP2. The ORFs of PBoV2-A6 were 703 aa for NS1, 221 aa for NP1 and 704 aa for VP1/VP2. The possible splicing of bocavirus NS1 transcripts recently shown to extend the length of NS1 proteins was not investigated here [6], [57].

### Prevalence of PBoV1 and PBoV2

Nucleic acids were extracted from 340 porcine stool samples. PBoV1-H18 related sequences were screened for using nested PCR with primers amplifying a 530-bp fragment of the NP1/VP1 region. The electrophoretic bands of the expected size were subcloned and sequenced. The results showed that the prevalence of PBoV1-H18 related viruses was high in China with 215 out of 340 (63.2%) porcine samples positive (Table 1). All PBoV1-H18 related sequences showed >99% identity with each other. For PBoV2-A6 nested PCR, 219 out of 340 (64.4%) samples were positive (Table 1), with the amplicons showing 90% to 100% identity with each other. 133 out of 340 (39.1%) samples were co-infected with both PBoV1-H18 and PBoV2-A6 related viruses (Table 1).

### Phylogenetic analysis of PBoV1 and PBoV2

**Figure 2:**
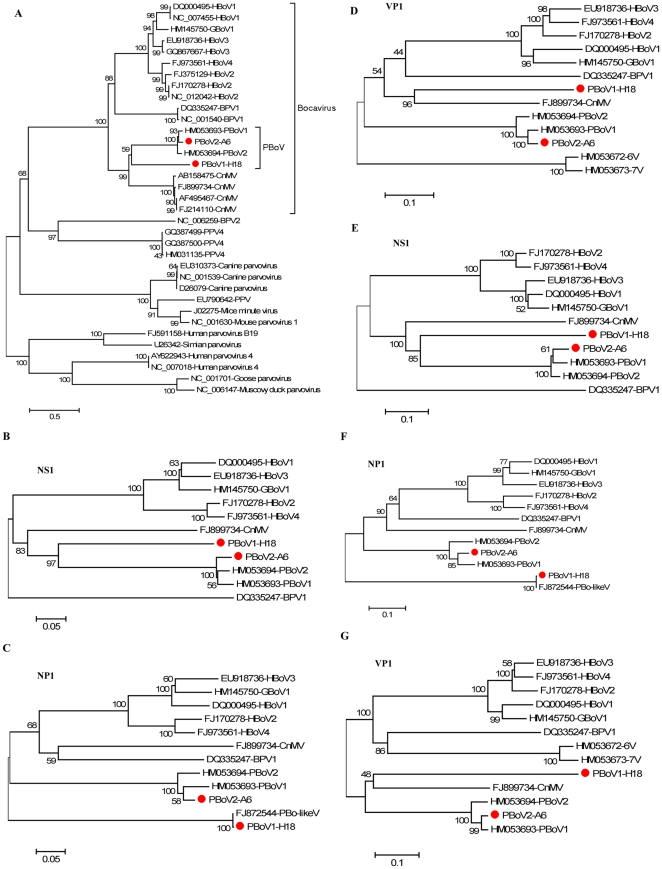
Phylogenetic tree constructed by the neighbor-joining method with 1,000 bootstrap replicates using MEGA4.0 software. Bootstrap values are indicated at each branching point. Scale bar indicates estimated genetic distance. Phylogenetic analysis of the nearly full-length genomes of PBoV1 and PBoV2 and 33 representative bocavirus species/strains (A). Phylogenetic analysis of nucleic acid and amino acid of NS1 (B and E), NP1 (C and F) and VP1 (D and G) ORFs of PBoV1-H18 and PBoV2-A6 and representative bocavirus species.

To determine the genetic relationship of PBoV1-H18 and PBoV2-A6 with recently described porcine bocaciruses and bocaviruses from other host species, both nucleotide and amino acid alignments were generated and used for phylogenetic analyses. When the whole genomes were considered porcine bocaviruses as a group (except for the V6/V7 variants with only NP1 sequences available), were most closely related to the canine bocavirus CnMV (Figure 2A). Phylogenetic analyses of the 3 ORFs – NS1, NP1 and VP1/VP2 – were also performed (Figure 2B–G). In all three regions, PBoV1-H18 was most closely related to the Chinese PBoV1 and PBoV2 recently reported by Cheng et al [28]. PBoV2-A6 was closely related in NP1 to the first reported partial porcine bocavirus sequence PBo-likeV from Sweden, the only region available for comparison (Fig. 2C, F) [27]. Table 2 numerically shows the protein similarities between PBoV1-H18 and PBoV2-A6 and other porcine and non-porcine bocaviruses.

**Table 2:** Comparison of pairwise amino acid distances (p-distance) of three genes between PBoV1-H18, PBoV2-A6 and other bocaviruses.

	PBoV1-H18	PBoV2-A6	PBo-likeV	PBoV1	PBoV2	6V	7V	HBoV1	HBoV2	HBoV3	HBoV4	GBoV1	CnMV	BPV1
PBoV1-H18														
NS1	-	51.8	-	52.9	51.7	-	-	59.6	58.6	59.4	59.2	59.7	55.8	65.8
NP1	-	62.3	0	64.2	62.2	-	-	67.9	67.6	68.7	66.2	68.8	64.8	67.6
VP1	-	52.3	-	52.0	52.7	63.1	63.4	61.8	62.8	62.4	62.7	62.3	53.6	63.2
PBoV2-A6														
NS1	51.8	-	-	6.8	5.8	-	-	58.7	58.3	60.1	58.3	62.2	55.9	66.7
NP1	62.3	-	62.3	7.9	10.1	-	-	54.7	58.1	55.1	56.7	57.5	51.9	58.1
VP1	52.3	-	-	2.7	7.1	59.2	59.5	52.1	53.1	53.7	52.7	53.5	46.9	55.0

The partial VP1 sequence of PBoV2-A6 related variants from different farms was also phylogentically analyzed and fell into two major clades we named PBoV2 genotype 1 and 2 (PBoV2-G1 and PBoV2-G2). The two previously described Chinese PBoV1 and PBoV2 “species” grouped within these two genotypes.

## Discussion

PBo-likeV was originally found in swine with postweaning multisystemic wasting syndrome (PMWS) in 2009 when approximately 35% of its genome sequence was reported [27]. The nearly full genomes of two distantly related porcine bocaviruses labeled PBoV1 and PBoV2 as well as two partial genomes labeled V6 and V7 were then reported in 2010 [28]. These bocaviruses grouped into 3 phylogenetic clades containing PBo-likeV, PBoV1/PBoV2, and V6/V7. In the present study, we characterize two nearly complete bocavirus genomes, one of which (PBoV1-H18) grouped with the PBo-likeV clade while the second (PBoV2-A6) fell with the PBoV1/PBoV2 clade. We provisionally named the genome from the H18 sample PBoV1-H18 since its closest homologue, PBo-likeV, was the first reported porcine bocavirus [27]. The virus from sample A6 was provisionally named PBoV2-A6 since it phylogenetically groups with the second reported set of porcine bocaviruses containing both PBoV1 and PBoV2 [28]. No close homologues of the V6/V7 clade were identified in this study. Under this proposed classification scheme, the viruses labeled PBoV1 and PBoV2 by Cheng et al, therefore both belong to the PBoV2 clade, the second reported clade of porcine bocaviruses [28]. Under this proposed taxonomic classification, the V6 and V7 bocaviruses [28] belong to the still only partially characterized PBoV3 clade.

**Figure 3:**
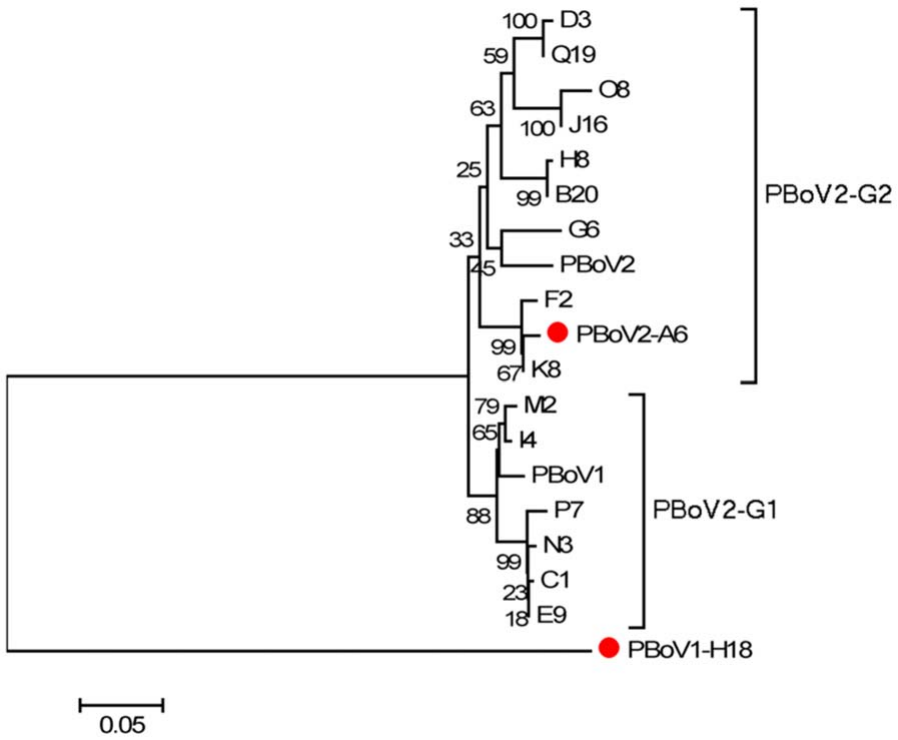
Phylogenetic tree of PBoV2-A6 related isolates in China showing the presence of two genotypes using a 439-nt VP1 gene sequence alignment. Bootstrap value was obtained from 1000 re-samplings of the data. The PBoV1-H18 strain is included as outgroup. Scale bar indicates estimated genetic distance.

Sequence analysis of the PBoV2 clade showed that their VP1/VP2 genes were highly diverse and could be classified into two genotypes (Figure 3). The partial NP1 and VP1 genes of PBoV1-H18 related viruses detected in this study were more highly conserved (99–100% identity), consistent with a recent report by Zhai et al reporting PBoV1 in Chinese pigs using partial VP1/VP2 nested PCR and sequencing [58]. Zhai et al also found a high prevalence of PBoV1 (69% in weanling piglets) with a higher frequency of PBoV1 in animals also infected with PCV2, PRRSV, PTTV or CSFV and in pigs with respiratory symptoms versus healthy pigs [58].

Members of the bocavirus genus contain an 3^rd^ ORF of unknown function labeled NP1 gene. Recently another parvovirus (PPV4) was identified in porcine feces that also contained a central 3^rd^ ORF, although unrelated in sequence to the bocaviruses NP1 [59]. None of the ORFs of PPV4 clustered phylogenetically with the bocaviruses (data not shown) but instead clustered with members of the parvovirus genus [59]. PPV4 is therefore unrelated to the bocaviruses reported here.

HBoV1 has been associated with respiratory symptoms while other HBoV may be associated with diarrhea and acute flaccid paralysis [5], [8]–[10], [11]–[22], [25]. A gorilla bocavirus was detected in captive animals in the US experiencing severe diarrhea [6]. PBoV1 was found in pigs with PMWS in Sweden [27] and in pigs with respiratory tract symptoms in China [58]. In the present study, both PBoV1 and PBoV2 were highly prevalent in both asymptomatic swine from five provinces of China. Further studies are needed to examine possible associations between infections with these different porcine bocaviruses, the viral loads excreted, the presence of co-infections and various porcine diseases.
